# Clock-dependent and system-driven oscillators interact in the suprachiasmatic nuclei to pace mammalian circadian rhythms

**DOI:** 10.1371/journal.pone.0187001

**Published:** 2017-10-23

**Authors:** Karine Abitbol, Ségolène Debiesse, François Molino, Pietro Mesirca, Isabelle Bidaud, Yoichi Minami, Matteo E. Mangoni, Kazuhiro Yagita, Patrice Mollard, Xavier Bonnefont

**Affiliations:** 1 Institut de Génomique Fonctionnelle, CNRS, INSERM, Montpellier, France; 2 Laboratoire Charles Coulomb, Université de Montpellier, CNRS UMR 5221, Montpellier, France; 3 Department of Physiology and Systems Bioscience, Kyoto Prefectural University of Medicine, Kyoto, Japan; Kent State University, UNITED STATES

## Abstract

Circadian clocks drive biological rhythms with a period of approximately 24 hours and keep in time with the outside world through daily resetting by environmental cues. While this external entrainment has been extensively investigated in the suprachiasmatic nuclei (SCN), the role of internal systemic rhythms, including daily fluctuations in core temperature or circulating hormones remains debated. Here, we show that lactating mice, which exhibit dampened systemic rhythms, possess normal molecular clockwork but impaired rhythms in both heat shock response gene expression and electrophysiological output in their SCN. This suggests that body rhythms regulate SCN activity downstream of the clock. Mathematical modeling predicts that systemic feedback upon the SCN functions as an internal oscillator that accounts for *in vivo* and *ex vivo* observations. Thus we are able to propose a new bottom-up hierarchical organization of circadian timekeeping in mammals, based on the interaction in the SCN between clock-dependent and system-driven oscillators.

## Introduction

The suprachiasmatic nuclei (SCN) of the hypothalamus lie at the top of the mammalian circadian system. They contain the central circadian pacemaker that regulates daily rhythms in physiology and behavior, and synchronizes subordinated circadian oscillators throughout the body [1]. A small set of so-called circadian clock genes drives the cell-autonomous rhythm in electrical activity of SCN neurons [2–4], coordinated within a SCN-wide intercellular network [5–7]. Daily resetting by external rhythms keeps the SCN exactly in tune with the 24-hour period of the outside world. By contrast, whether internal body rhythms are able to entrain and regulate the pace of SCN oscillations is less clear.

Importantly, the SCN molecular clockwork is resilient to major systemic rhythms, such as daily variations in glucocorticoids and core body temperature, which is thought to prevent interference with environmental resetting [8]. Both glucocorticoid signaling and temperature are well-characterized synchronizing cues of peripheral circadian oscillators [8–10]. Yet the glucocorticoid receptor is barely expressed in the SCN, and injections of dexamethasone, a glucocorticoid hormone analogue, is unable to phase shift circadian gene expression in the SCN [11]. Moreover, neither heat pulses nor temperature cycles are able to reset the rhythmic expression of a clock gene reporter in cultured SCN from adult mice [8, 12]. Remarkably, such resilience to systemic feedback may be a unique property of the intact mature SCN network since temperature has been shown to entrain circadian rhythms in the juvenile SCN [13] or when neural communications are interrupted [8].

However, this view has been challenged when other readouts of the SCN pacemaker were investigated. Several studies have shown that the endogenous rhythm in glucocorticoids interferes with photic entrainment of SCN-driven rhythmicity, in diurnal as well as nocturnal animals, probably through indirect pathways [14–17]. Likewise, subtle increases in temperature are sufficient to phase shift rhythmic electrical activity within SCN slices from adult rats [18]. Other peripheral factors, such as the fasting-induced hepatokine FGF21, alter circadian behavior through direct effect at the SCN level, but without noticeable change in hypothalamic circadian clock gene expression [19]. Hence systemic feedback at the SCN level remains ill-understood and has been the matter of a longstanding debate [17, 20].

In the present study, we address this issue and investigate how impaired systemic rhythmicity translates to the SCN of lactating mice that exhibit reduced daily amplitude in both corticosterone [21] and body temperature [22] rhythms. We find an intact molecular clockwork in the SCN of dams, but suppressed rhythms in system-driven gene expression and electrophysiological activity. The experimental findings, together with mathematical modeling, strongly suggest that body rhythms likely regulate SCN oscillations downstream of the circadian clock.

## Materials and methods

### Animals

All experiments were approved by the Animal Welfare and Ethical Review Body of Languedoc-Roussillon (CEEA-LR-12113) and the Kyoto Prefectoral University of Medicine Ethical Committee (KPUM 22–56, M26-206). All efforts were made to minimize suffering.

C57BL/6JRj mice (Janvier labs) were housed in ventilated cabinets (Tecniplast) at 23±1°C, with free access to food and water, in a 12-hour light 12-hour dark photoperiod (lights on defined as Zeitgeber Time 0, ZT0), except when specified otherwise. Virgin females were housed either individually to produce age-matched controls or with males for one week and isolated until experiment at mid-lactation. All the litters were normalized to 6 pups, and dams were used for electrophysiological and gene expression measurements at mid-lactation (days 8–12), when interaction with the pups remains high, as assessed by their elevated prolactin levels [23].

### Telemetric measurement in freely-moving mice

This experiment was conducted longitudinally, along a complete reproductive cycle in the same animals. Telemetric transmitters (TA10EA-F20, Data Sciences International) were implanted into a subcutaneous pocket on young adult female mice under anesthesia. Advil (7 ml/l) was added to the drinking water for 4 days post-surgery in order to manage possible pain. Measurements of body temperature, general activity and heart rate started 10 days after recovery from the surgical implantation, using a telemetry receiver and an analog-to-digital conversion data acquisition system for display and analysis by the Dataquest^™^ ART^™^ software (Data Sciences International). Recordings were performed for 4 consecutive days in the virgin condition, stopped when a male was added, and resumed when females were obviously pregnant, and continued throughout lactation and after weaning.

### Monitoring of running-wheel activity

Mice were housed in cages equipped with a running wheel. Voluntary activity was measured as running wheel revolutions recorded in one-minute bins and analyzed with the ClockLab software (Actimetrics).

### In vitro electrophysiology

Lactating and control mice were killed by decapitation around ZT1 and ZT13 for daytime (ZT2-8) and night-time (ZT14-21) recording, respectively. Coronal brain slices (250 μm thick) containing the SCN were prepared using a vibratome, in cold artificial cerebrospinal fluid (ACSF, containing in mM: 125 NaCl, 5 KCl, 1 NaH_2_PO_4_, 1 MgSO_4_, 2.5 CaCl_2_, 24 NaHCO_3_, 15 glucose, oxygenated with 95% O_2_, 5% CO_2_, pH 7.4), and were allowed to recover for at least 1 hour at room temperature. Experiments were conducted at 36°C, using an EPC9 patch-clamp amplifier (HEKA Elektronik) in the loose-patch or whole-cell configuration. Recordings with R_series_ higher than 40 MΩ or with holding currents larger than 100 pA (at -60 mV) were discarded.

In the loose-patch configuration, glass pipettes (6–8 MΩ) were filled with ACSF. Fast current transients reflecting spikes were recorded at a holding potential of 0 mV for at least one minute. Unstable traces were excluded from analysis.

In whole-cell configuration, pipettes (6–8 MΩ) contained an intracellular solution (in mM): 140 KCl, 4 NaCl, 10 HEPES, 5 EGTA, 1 MgCl_2_, 0.5 CaCl_2_, 5 Phosphocreatine, 1 Na-GTP, 5 Mg-ATP, pH 7.3, osmolality adjusted to 290–300 mOsM. Recordings were performed within the first two minutes after break-in to prevent artifacts due to dialysis.

All data were processed with the Igor Pro software (WaveMetrics, Inc). Daytime and night-time distributions of events were compared using the Kolmogorov-Smirnov test in Prism (GraphPad Software, Inc).

### Real-time quantitative RT-PCR and analysis

SCN were rapidly dissected [24], frozen in liquid nitrogen and stored at -80°C. The two SCN from two mice were pooled, and 4 pooled samples were proceeded per condition at each time point. Total RNA samples were isolated using the RNeasy microkit (Qiagen) and treated with DNase (Qiagen). Reverse transcription was performed using random primers and SuperScript III (Invitrogen). Quantitative PCR was performed using 2× SybrGreen Mix and LC480 Real-Time PCR System (Roche). Relative expression levels were calculated by the ΔCt method, using reference genes (*Hprt2*, *Gapdh* and *Trfr1*) selected with the GeNorm procedure [25]. Primer sequences are given in S1 Table.

Gene expression profiles were analyzed with both the parametric cosinor procedure and the nonparametric JTK-CYCLE algorithm [26]. For cosinor analysis, a 24-hour cosine curve was fitted to time series of mRNA levels in Prism (GraphPad Software, Inc), and an extra sum-of-squares F test was used to test whether the amplitude was similar in virgin and lactating conditions. This hypothesis was rejected for p < 0.05. JTK-CYCLE calculated the minimum false discovery rate at which a gene is mistakenly called cyclic (Q value) in a global analysis of all gene expression patterns.

### PER2::LUC imaging

Bioluminescence imaging was performed as previously described [27]. PERIOD2::LUCIFERASE mice [1] were killed at ZT8-12 and their brain quickly removed, and transferred to ice-cold Hank’s balanced salt solution oxygenated with 95% O_2_, 5% CO_2_. Coronal SCN sections (200 μm thick) were set on a culture membrane (Millicell-CM; Merck Millipore) in a sealed Petri dish (diameter 35 mm) with 1.2 ml of DMEM supplemented with 15 mM HEPES, 1.2 g/l NaHCO_3_, 20 mg/l kanamycin, 5 μg/ml insulin, 100 μg/ml human transferrin, 100 μM putrescine, 20 nM progesterone and 30 nM sodium selenite, and 0.2 mM of beetle luciferin. PERIOD2::LUCIFERASE imaging was performed using an integrated incubator-microscope system (LV100 and LV200; Olympus, Tokyo, Japan). Time-lapse luminescence images were taken every hour with 59 min exposure duration. Signal intensity in region of interests was analyzed using AquaCosmos software (Hamamatsu Photonics, Hamamatsu, Japan). Data were detrended by 24-hr moving average.

### Mathematical modeling

The double regulation of the average neuronal firing in the SCN, N(t), by circadian clock oscillations, C(t), on the one hand, and a systemic feedback K(t), on the other hand, was considered. We constructed a minimal model with the following building blocks:

1. N(t) spontaneously reaches a level N_max_, with a kinetic parameter k_N_;
2. C(t) oscillates with a fixed independent period close to 24 hours, and promotes oscillations of N(t) below N_max_, through a coupling constant k_C_;
3. K(t) facilitates the interaction between C and N with a fixed delay τ, and a threshold-like behavior: the effect is weak below a value K_0_, and maximum above it. A minimum coupling value, ε, exists when K is null. K(t) was not explicitly modeled, being directly driven by N(t).

This naturally leads to a Hill-like excitatory dynamics for N(t), in the following form:

$$\frac{dN}{dt}=-k_{C}\frac{{\left(\frac{K\left(t-\tau \right)}{K_{0}}\right)}^{n}+\varepsilon }{1+{\left(\frac{K\left(t-\tau \right)}{K_{0}}\right)}^{n}}C\left(t\right).N\left(t\right)+k_{N}\left(N_{max}-N\left(t\right)\right)$$ (1)

In addition, we implemented the resetting effect of behavioral arousal [28]. Locomotor activity, L(t), is oppositely related to SCN neuronal firing (L(t) = N_max_-N(t)), and resets C(t) to its maximal value when dL/dt crosses a simple positive threshold.

For illustrative purposes, we used the following numerical values in all the simulations: k_C_ = 0.05; k_N_ = 0.01; ε = 0.15; K_0_ = 0.2; n = 25; N_max_ = 1. The values of τ and the period of C(t) were determined from the literature, as described in the text. The dynamics was integrated using an Euler scheme in MATLAB.

## Results

### Lactating mice exhibit altered daily rhythms in behavior and physiology

**Fig 1:**
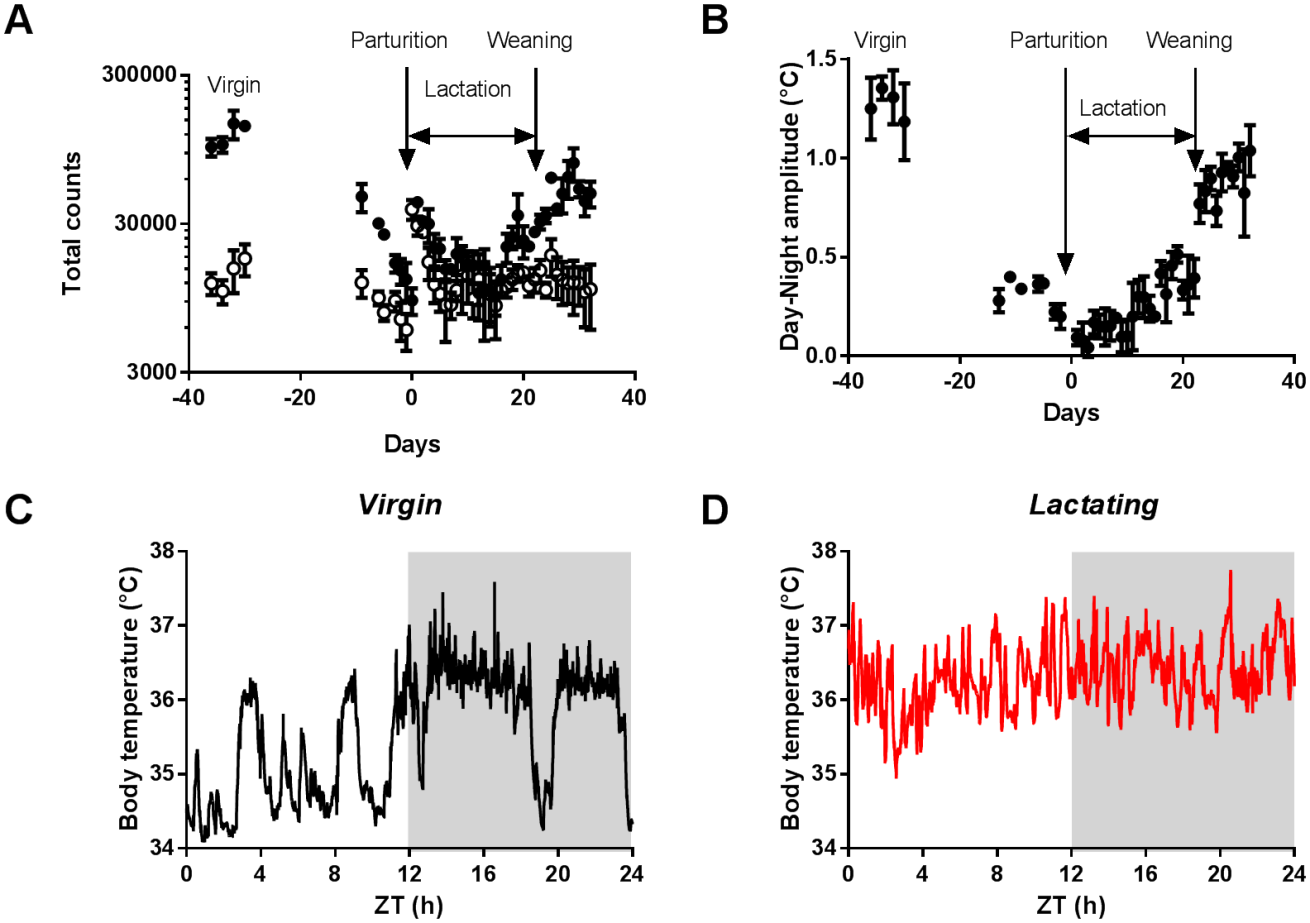
Longitudinal telemetric monitoring of locomotor activity and body temperature in female mice. A. Daytime (open circles) and night-time (closed circles) counts in locomotion throughout a reproductive cycle. B. Average day-night amplitude in body temperature throughout a reproductive cycle. (mean±sem, n = 3 mice; temperature amplitudes were significantly smaller every days of lactation as compared to the period before mating, F(21, 42) = 9.333, p<0.0001 2-way ANOVA Tukey's multiple comparisons test). C and D. Representative example of 24-hour variations in body temperature, recorded in a same mouse before mating (C) and during lactation (D). ZT0 defined as time of lights on.

**Fig 2:**
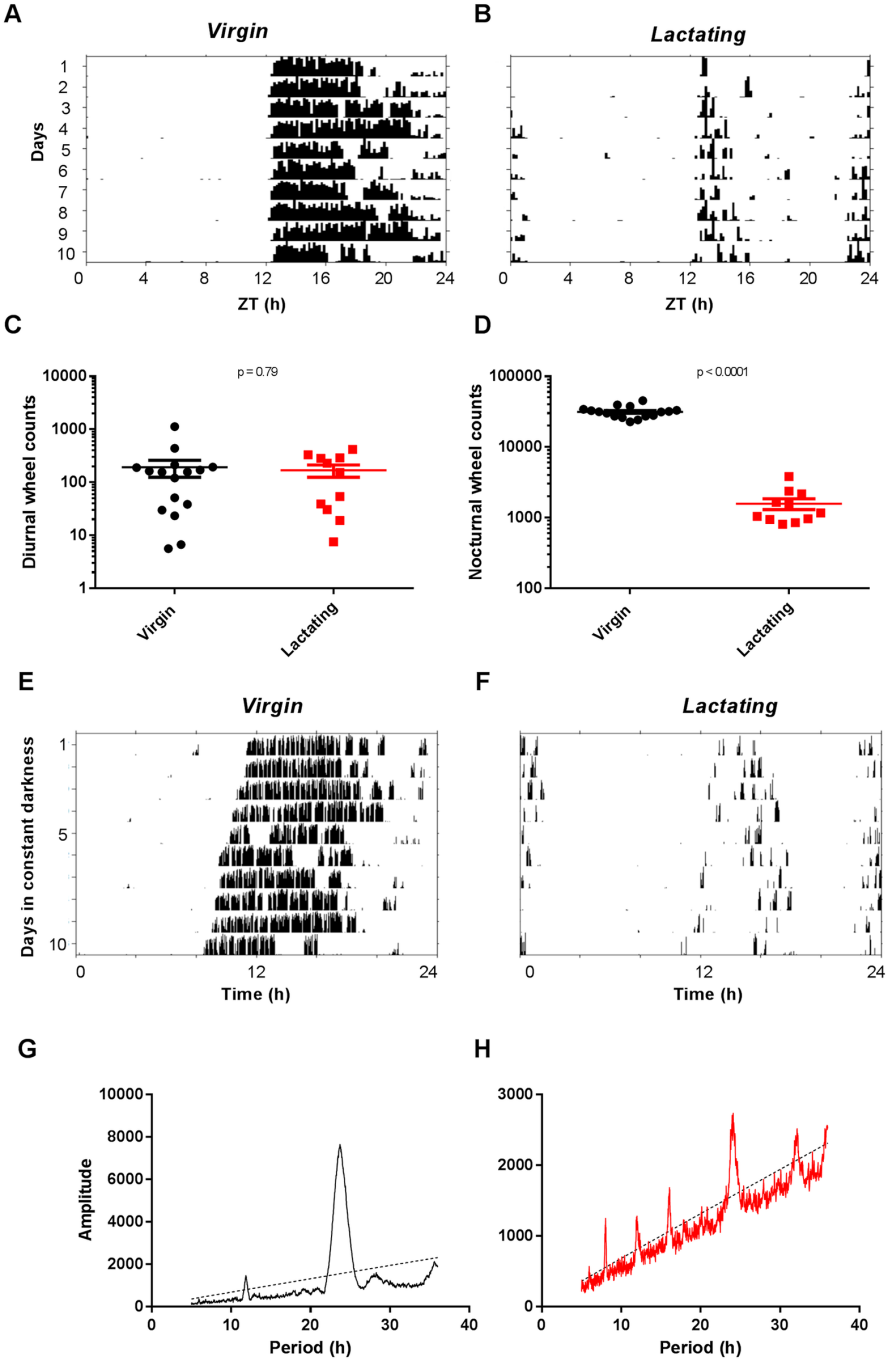
Voluntary running-wheel behavior in virgin and lactating mice. A-D. Reduced activity during lactation, under light-dark conditions. Representative actograms from virgin (A) and lactating (B) female mice. Quantification of wheel counts during daytime (C) and night-time (D). Differences were considered significant for p < 0.05, two-tailed unpaired t-test. E-H. Noticeable rhythmic organization of behavior under constant darkness. Representative actograms (E and F) and corresponding periodograms (G and H) showing a major power peak in the circadian range for both virgin (E and G) and lactating (F and H) females (the calculated free-running period was 23.72±0.01 hr and 23.77±0.14 hr, for virgin and lactating females, respectively, p>0.71 two-tailed unpaired t-test, n = 4 mice for each condition). Note that low levels in wheel counts and the presence of pups in the cage prevent the measurement of a reliable free-running period for lactating mice.

Longitudinal monitoring of female mice implanted with telemetric probes showed that daily rhythmicity of both home cage activity and body temperature was sharply decreased during lactation. Nocturnal locomotion dropped to daytime levels from the end of gestation until the last week of lactation, except for a well-characterized episode of hyperactivity following parturition (Fig 1A). Despite reduced activity, dams exhibited continuously elevated body temperature, with the average day-night amplitude remaining below 0.5°C for at least two weeks post-partum (Fig 1B, 1C and 1D). This is likely due to their limited ability to dissipate heat under conventional husbandry conditions [22]. Lactating mice also exhibited altered rhythms in voluntary running-wheel activity, with decreased wheel counts at night (Fig 2A). However, a free-running rhythm in activity persisted under constant darkness (Fig 2B), suggesting that circadian timekeeping remained largely functional in dams.

### Clock-driven but not system-dependent molecular rhythms persist in the SCN of lactating dams

**Fig 3:**
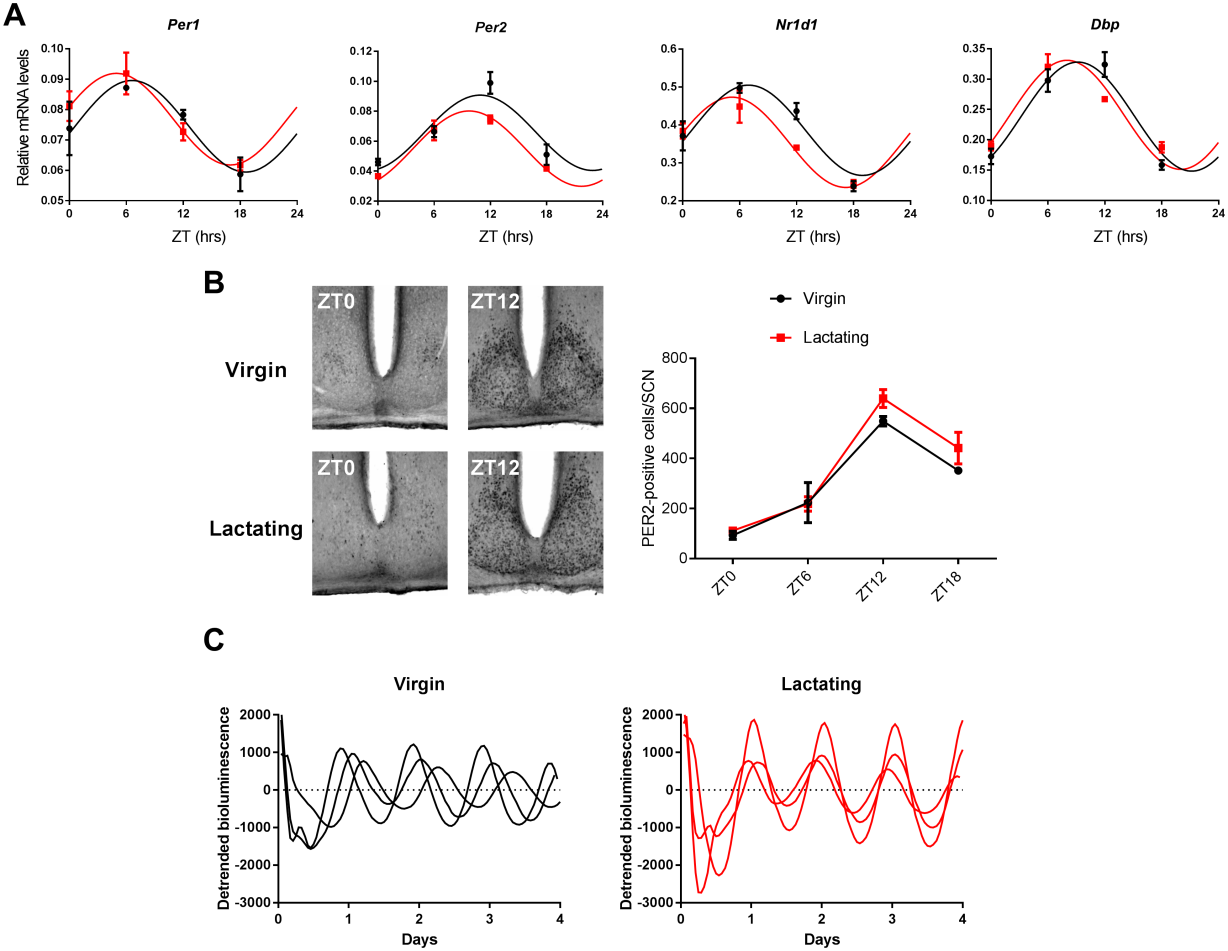
The circadian clockwork is preserved in the SCN of lactating mice. A. Expression profiles of circadian clock and clock-related genes in the SCN of virgin (black) and lactating (red) mice, as assessed by quantitative PCR (mean ± SEM, n = 4 samples for each time point. The sine lines represent the best cosinor fit for each dataset (see also Table 1) B. Representative micrographs (left panels) of immunostaining for PER2 in the SCN from virgin (top) and lactating (bottom) mice, at ZT 0 and ZT 12. The number of PER2-immunopositive cells per SCN was quantified over a complete daily cycle (right panel, mean ± SEM, n = 3 mice for each time point). A highly significant time effect was observed (p < 0.0001), with no difference between reproductive states (p = 0.12, two-way ANOVA). C. Representative recordings of PER2::LUC expression showing robust circadian oscillations in SCN slices from both virgin (left panel) and lactating (right panel) females. The time of occurrence of the circadian oscillation peak during the interval between 12 and 36 hours in culture, did not differ between both conditions (25.89±0.97 hrs, n = 8, and 24.87±0.45 hrs, n = 7, respectively, p = 0.17, unpaired t-test).

To explore the functionality of the circadian pacemaker in lactating mice, we investigated the molecular clockwork in their SCN. Levels of major clock-related mRNA (Fig 3A and Table 1) and of the PER2 protein (Fig 3B) did not differ between the SCN from virgin and lactating females. Moreover real-time monitoring of the PER2::LUC circadian reporter in SCN slices [1] revealed self-sustained oscillations in bioluminescence emission, irrespective of the reproductive status of the donor mouse (Fig 3C). These results indicate that the circadian clock is not impaired during lactation and is fully able to drive molecular rhythms in the SCN.

**Table 1:** Analysis of daily expression patterns of clock-related genes and HSF1-target genes in the SCN of virgin and lactating mice.

Gene	Female status	JTK_CYCLE		Cosinor analysis	
		Q value	Min and max amplitude (10^−4^)	Goodness of fit (R^2^)	Amplitude (Virgin vs Lactating)
*Per1*	V	0.0027	68–189	0.55	p = 0.79
	L	0.0001	59–202	0.66	
*Per2*	V	0.0007	121–361	0.73	p = 0.38
	L	0.0001	171–283	0.85	
*Nr1d1*	V	0.0001	656–1704	0.80	p = 0.25
	L	0.0001	341–1389	0.73	
*Dbp*	V	0.0105	804–1264	0.85	p = 0.09
	L	0.0002	521–884	0.81	
*Hspa1a*	V	0.0078	15–31	0.66	*, p = 0.02
	L	0.0058	8–16	0.46	
*Odc1*	V	0.0140	144–512	0.57	**, p = 0.004
	L	0.7711	0–128	0.12	
*Chordc1*	V	0.0001	285–477	0.87	**, p = 0.008
	L	1	0–303	0.10	

Nonparametric analysis with the JTK_CYCLE algorithm (see Methods): note the increased ratio between amplitudes measured in the SCN of virgin (V) and lactating (L) mice. Q value is the minimum false discovery rate at which a gene is mistakenly classified as cyclic. Parametric analysis with the cosinor method (see Methods): the amplitudes of gene expression patterns were considered different between virgin and lactating mice for p<0.05, extra sum-of-squares F test.

**Fig 4:**
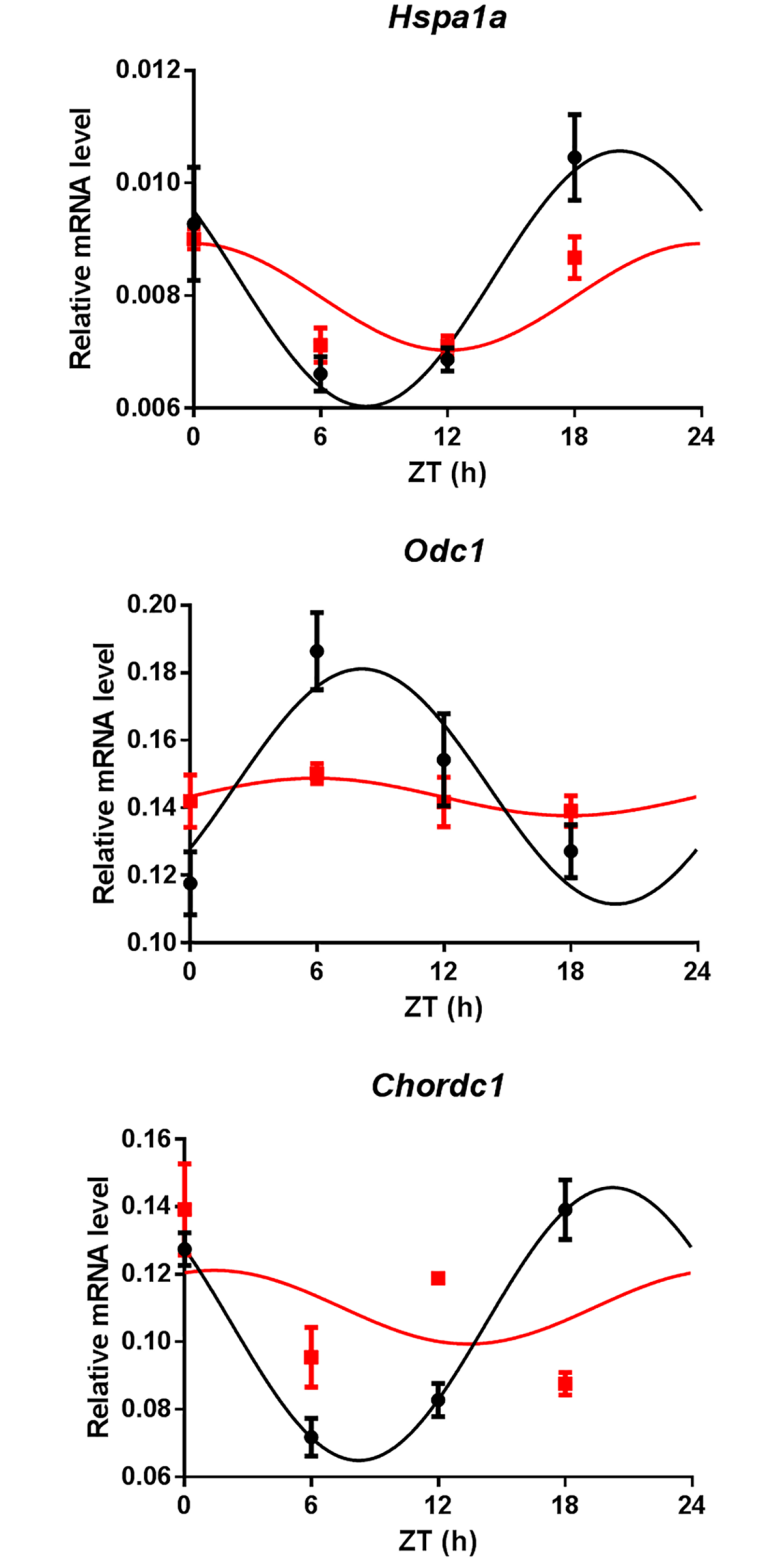
Altered rhythmic expression of putative HSF1-target genes in the SCN of lactating mice, (mean ± SEM, n = 4, same samples and analysis as for Fig 3A, see also Table 1).

We then questioned whether the expression of genes responsive to systemic signals could be altered in the SCN of lactating mice in spite of their preserved circadian machinery. We focused our attention on putative targets of the heat shock transcription factor HSF1 since genes of the heat shock pathway exhibit robust circadian expression in the SCN [29], and the rhythmic activation of HSF1 in peripheral tissues is driven by systemic cues, such as daily variations in core body temperature [10, 30, 31]. We first performed a cross-analysis between a genome-wide study of human HSF1 targets [32] and the circadian expression profiles database of the male mouse SCN [33]. Among the thirty top HSF1-bound human gene promoters, three mouse gene homologues (*Hspa1a*, *Chordc1* and *Odc1*) exhibited significant circadian expression in the murine database. We found that mRNA of all three genes followed a daily profile in the SCN of virgin females that was significantly dampened in lactating dams (Fig 4 and Table 1). Hence, the SCN contain clock-driven rhythms resilient to systemic perturbations, and molecular oscillators that are altered during lactation.

### Reduced daily rhythm in SCN neuronal firing during lactation

**Fig 5:**
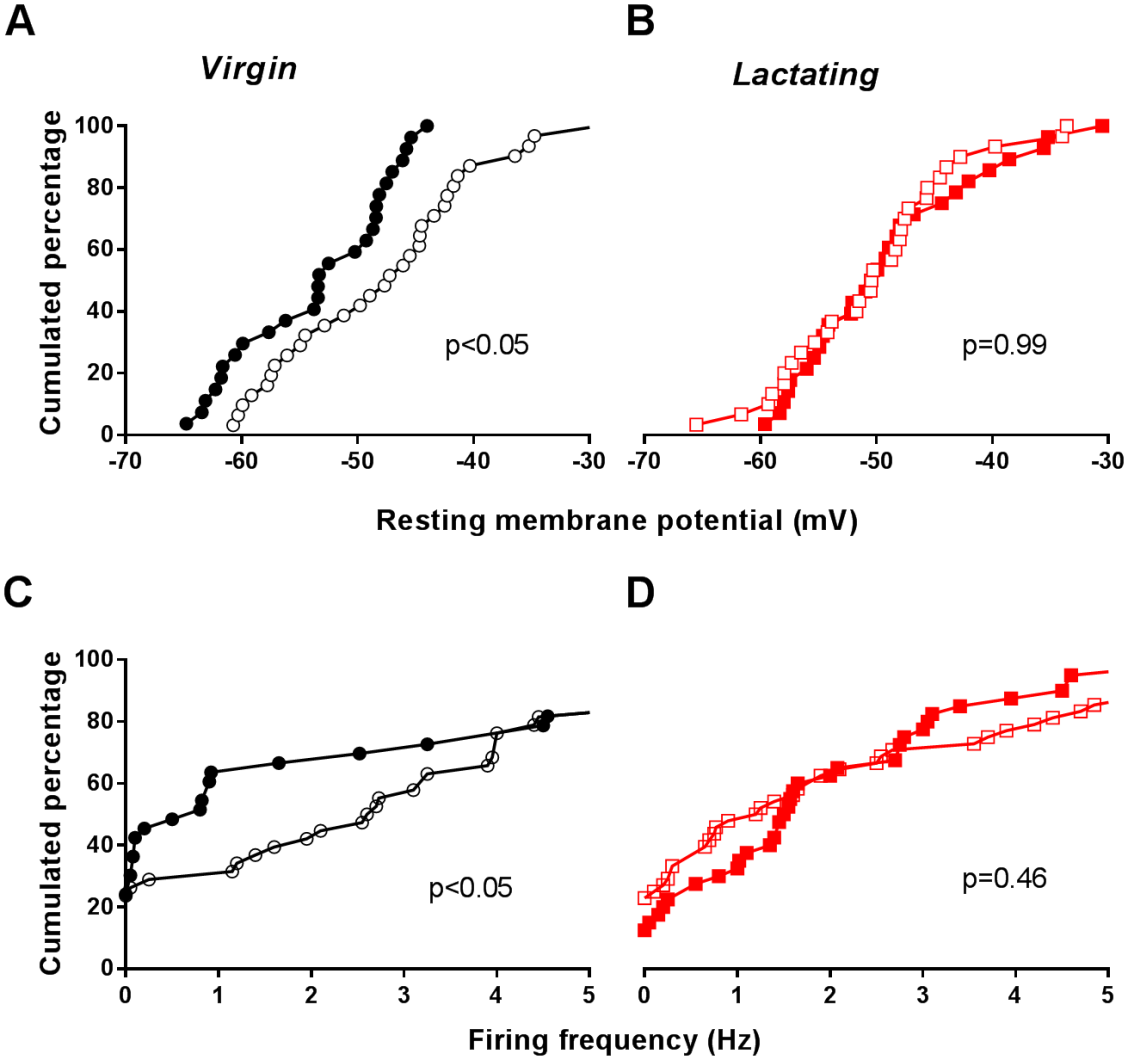
The daily rhythm in SCN electrical properties is suppressed in lactating mice. A-B. Cumulative distributions of membrane potentials measured in patch-clamped cells, from virgin (A) and lactating (B) mice. C-D. Cumulative distributions of extracellular single neuron firing frequencies, from virgin (C) and lactating (D) mice. SCN slices were recorded during either daytime (empty symbols) or night-time (filled symbols). The firing frequencies measured in patch-clamped cells are shown in S1 Fig. Differences were considered significant for p < 0.05, Kolmogorov-Smirnov test, n = 30 to 48 cells, from at least 5 different mice for each condition.

To investigate how this molecular duality of the SCN translates at the physiological level, we measured electrical activity of neurons in SCN slices, a known output of circadian clock genes [2–4, 34]. Remarkably, typical day-night differences in electrical properties observed in virgin females vanished during lactation. The suppression of daily variations in resting membrane potential in lactating mice (Fig 5A) was associated with overlapping daytime and night-time distributions of action potential discharge rates, as measured in two independent sets of experiments, using either whole-cell patch-clamp (S1 Fig) or extracellular monitoring of single-neuron activity (Fig 5B). Thus, lactation resulted in uncoupling of electrical firing from the circadian clockwork in SCN neurons.

### Mathematical modeling of a systemic feedback upon the SCN

**Fig 6:**
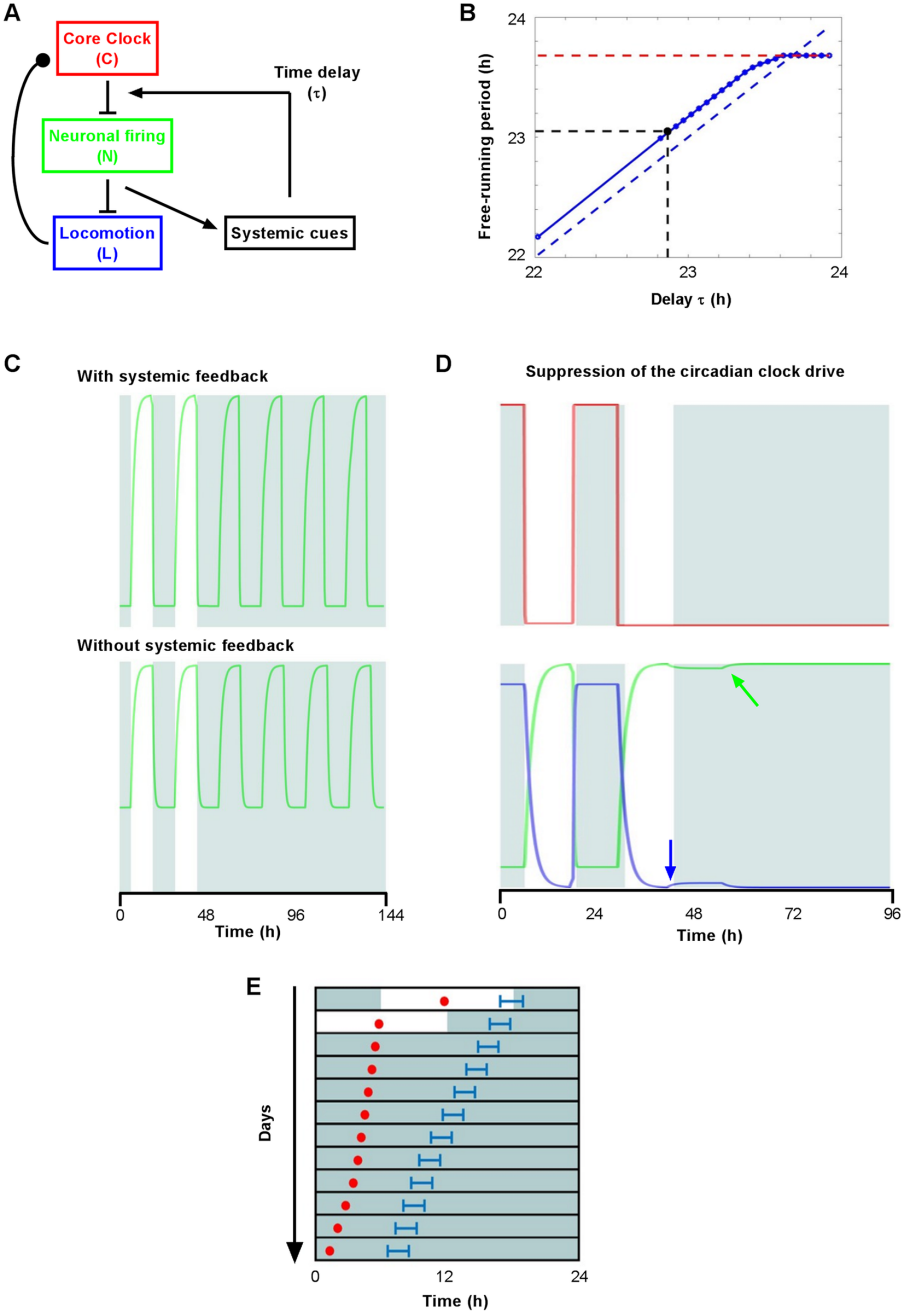
Modeling the regulation of SCN outputs by systemic feedback. A. Diagram depicting functional interactions entered into the mathematical model. B. The apparent free-running period of the locomotor rhythm (solid blue line) depends on the values of t and of the intrinsic period of the circadian clock. This example is constructed with numerical values published by Reinke et al. [31], with the clock period set at 23.68 h (dotted red line). For an apparent period of 23.05 h, the value of t is 22.86 h (dotted black lines). The dotted blue line represents the line y = x. C. Predicted rhythmicity of the SCN electrical output when systemic feedback is suppressed, as in SCN slices. The reduced amplitude of the SCN output recapitulates dampened rhythms observed in lactating and *Hsf1-/-* mice [35]. D. The suppression of clock oscillations (red line) recapitulated the rebound-shape pattern of SCN firing (green arrow) and pre-dark locomotor behavior (blue arrow) observed in Cry1-/- Cry2-/- mice [2, 36]. E. Phase-dissociation between clock oscillations and overt rhythms after abrupt phase-shift of C, as in the case of a 6-hour advance of the light schedule [37]. (Red dots and blue lines represent the middle of the up-state of C and the onset of L, respectively.)

Taken together, our data suggest that systemic cues in lactating dams alter SCN output rhythms downstream of the clock, through a clutch-like effect. We investigated this hypothesis further using a minimal model in which the coupling between SCN clock oscillations (C) and the average SCN rhythm in neuronal firing (N) is facilitated by a rhythmic SCN output. We implemented an unknown time delay (τ) to account for the physiological and molecular processing of this systemic feedback loop (Fig 6A, see Methods for mathematical details).

Remarkably, systemic regulation proved to be a critical determinant of the frequency of SCN outputs. The apparent period of locomotor activity (L) was a complex mix between the intrinsic period of C and the kinetics of the systemic feedback, computed in τ (Fig 6B). We took advantage of this relationship to estimate a biologically-relevant value of τ, by comparing the free-running periods reported from *Hsf1+/+* and *Hsf1-/-* mice [31]. We reasoned that the intrinsic period of their circadian clock would be given by the apparent period measured in *Hsf1-/-* mice (23.68 hr), assuming that the SCN would be resilient to systemic feedback in the absence of HSF1. We then calculated that τ = 22.86 hr accounts for the period (23.05 hr) of *Hsf1+/+* littermates (Fig 6B). Hence, in this framework the systemic feedback loop functions as an hourglass mechanism with an intrinsic period in the circadian range, which we termed Klepsydra (K), from the Greek word for the water-clock used during Antiquity.

Then we explored other predictions of our model using the theoretical value of τ, as calculated above. First, to assess the functional relevance of Klepsydra, we suppressed its influence in the model (K = 0), as this might occur in SCN slices cultured under controlled constant conditions. Accordingly, the clock alone was sufficient to drive robust circadian output of the SCN (Fig 6C), although with reduced amplitude as observed in lactating mice and *Hsf1-/-* mice [35]. Conversely, we investigated the effect of Klepsydra alone by removing the clock drive from the model (C = 0), such as in animals devoid of a functional clock (e.g. *Cry1-/-Cry2-/-* mice). This action completely suppressed circadian oscillations of SCN outputs under constant conditions, with constitutively high neuronal firing and low locomotor activity (Fig 6D). It however unmasked a brief system-dependent decrease in neuronal firing that gave rise to a short bout of locomotor activity around the simulated light offset (Fig 6D).

Finally, to investigate the relative strength of the circadian pacemaker and Klepsydra, we imposed an abrupt phase-shift to the SCN clock. This simulation showed that the SCN clock remained locked to its new phase angle, thus highlighting its resilience to resetting by systemic rhythms. On the contrary, the SCN output progressively aligned with the clock phase, leading to transient phase-dissociation between the clock and output (Fig 6E), as experimentally reported from rats released under constant darkness after a light-phase advance [37]. Thus, our model indicates that systemic feedback at the SCN level is coherent, and predicts that it contributes to regulating overt circadian rhythms.

## Discussion

Altogether our data show how systemic feedback confers robustness to circadian rhythms, without interfering with phase entrainment of the pacemaker itself. Using a lactating mouse paradigm, we were able to disclose the dual regulation of SCN daily oscillations by the circadian clock and systemic rhythms. The impaired rhythm in system-driven gene expression in the SCN of lactating mice indicates that physiological conditions impinge upon SCN rhythmicity. The unaltered SCN clock during lactation and suppressed daily variations in neuronal firing, a known output of circadian clock genes [2–4, 34], are in keeping with the resilience of the clock to peripheral rhythms [8, 11, 12], but suggest that systemic cues modulate SCN rhythms downstream of the molecular clockwork. This may explain how mammalian females retain functional circadian timekeeping, a pre-requisite for proper maternal behavior and milk production [38–43], while facing the day-and-night demand from their young.

Remarkably, this phenotype of lactating mice is consistent with previous data showing that overt rhythms but not the SCN clock are entrained by systemic cues [8, 11, 14, 17–19]. We built a simple mathematical model to test this apparent paradox: how can clock-driven systemic rhythms feedback at the level of the SCN and pace themselves without entraining the clock? This model not only validates the coherence of such a new organization of circadian timekeeping, but also supports a number of empirical data, as follows:

1. *Daily SCN outputs in lactating dams*. The simulation of dampened systemic rhythms acting at the SCN level recapitulated the reduced day-night amplitude in locomotor activity and SCN electrical firing, as observed in lactating mice despite their fully functional clock. It remains uncertain whether the phase and period of output rhythms are altered during lactation since these parameters are barely measurable in dams, because of low levels of running-wheel activity and the presence of the pups.
2. *The phenotype of Hsf1-/- mice*. The numerical suppression of the feedback loop in our model recapitulated the increased free-running period [31] and diminished amplitude [35] in wheel behavior observed in *Hsf1-/-* mice. This result indicates that HSF1 may partly mediate systemic regulation at the SCN level, and underscores the heat-shock pathway as a conserved capacitor of circadian rhythms since similar effects have also been reported in drosophila [44]. Future transcriptome analysis will establish to which extend HSF1 signaling contributes to Klepsydra, and what other cellular pathways are involved, such as FGF receptors for example [19]. In addition, as communications between SCN subdivisions modulate their entrainment by temperature [8], further studies will be necessary to decipher the respective roles of intracellular and intercellular signaling in the course of peripheral feedback.
3. *The residual rhythmicity in circadian clock-deficient mice*. Clock deletion in the model simulated the sustained high neuronal firing rate [2] and low level of locomotor output [45] reported from clock-deficient *Cry1-/- Cry2-/-* mice. It also recapitulated their unique rebound-shaped SCN activity [2] and pre-dark behavior [36] under light-dark conditions. This suggests that these features are actually fingerprints of systemic feedback upon the SCN.
4. *Uncoupling between SCN rhythms during jetlag*. Our phase shift simulation recapitulated the dissociation reported between the SCN clock and physiological output rhythms [37, 46]. It has long been known that the circadian pacemaker resets faster than measurable rhythms after an abrupt light phase shift [47, 48], although some divergence may exist among clock genes and SCN subdivisions [14, 49–52]. We show that a major source of inertia during jetlag eventually stems from systemic feedback upon the SCN. This suggests that an efficient method to accelerate re-entrainment after a light phase shift may reside in suitable alterations of peripheral rhythmicity [53].

Noteworthy, most of the criteria assigned to Klepsydra may be fulfilled by the night hormone, melatonin. Its rhythmic secretion by the pineal gland is controlled by the SCN [54] and, similarly to other peripheral cues such as glucocorticoids or temperature, it is known to phase shift SCN outputs [55]. However, melatonin is unable to entrain circadian clock gene expression in the SCN [56], which questions its chronobiotic action. The systemic feedback loop described here may provide mechanistic support for endogenous melatonin in stabilizing biological rhythms and, more importantly, for the resetting effect of exogenous melatonin, broadly used to fight against jetlag after transmeridian flights [57].

Another important prediction of our present study and model lies in the value of the time delay, τ, assigned to Klepsydra. As such, the interaction described here between clock-driven and system-dependent rhythms resembles the two-coupled circadian oscillators from the seminal model proposed to underlie the stability and lability of circadian rhythms [58]. The occurrence of systemic regulation of the SCN with a delay that falls in the circadian range is in keeping with the previous identification of other extra-SCN circadian oscillators, such as the food-entrained oscillator [59] or the methamphetamine-entrained oscillator [60]. However, whether Klepsydra encompasses any of these circadian oscillators remains an open question. In addition, it is worth noting that the value of τ may actually reflect the combination of multiple cue-specific time delays, gated by the time of day, as heat pulses [18] and melatonin [55] given at various time points produced different phase shifts of the SCN electrical rhythm.

In conclusion, this study unveils systemic feedback at the SCN level, which turns the classical top-down hierarchy of circadian timekeeping into a bottom-up organization that provides robustness and plasticity to biological rhythms. This new oscillatory mechanism underscores how specific systemic cues may shape mammalian daily rhythms without altering the SCN clock itself [53, 57]. This paves the way for novel treatments targeting the "weak link" hypothesized between the SCN clock and outputs during ageing and disease [4].

## Supporting information

S1 Fig Firing frequency in SCN neurons, as assessed by whole-cell patch-clamp. Related to Fig 5. Daytime (empty symbols) and night-time (filled symbols) distributions of firing frequencies in patch-clamped neurons in SCN slices from virgin (upper panel) and lactating (lower panel) female mice. These data were analyzed as explained in Fig 5.
(PDF)

S1 Table Sequence of qPCR primers. (DOCX)
