# Supplementary figures and images for: Clock-dependent and system-driven oscillators interact in the suprachiasmatic nuclei to pace mammalian circadian rhythms
Source: PLoS One. 2017 Oct 23;12(10):e0187001. doi: 10.1371/journal.pone.0187001 (PMC5653358; doi:10.1371/journal.pone.0187001)

Virgin

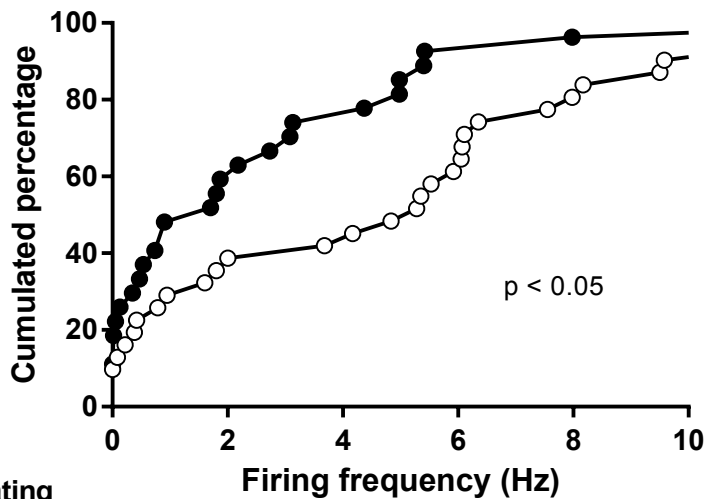

Lactating

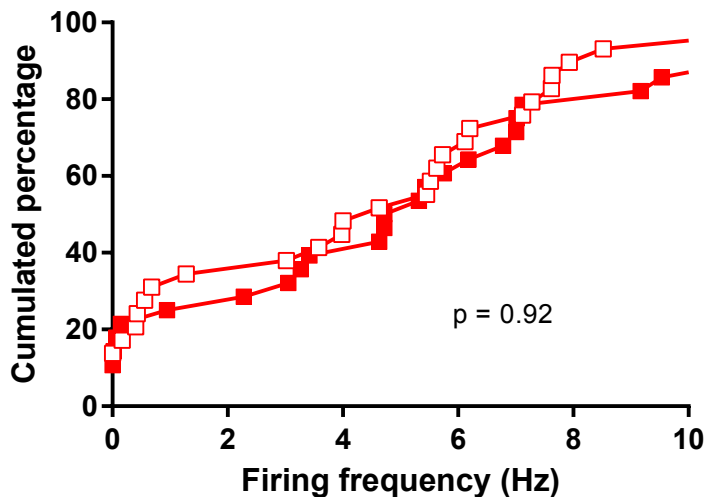

Supplement: S1 Fig — Related to Fig 5. Daytime (empty symbols) and night-time (filled symbols) distributions of firing frequencies in patch-clamped neurons in SCN slices from virgin (upper panel) and lactating (lower panel) female mice. These data were analyzed as explained in Fig 5. (PDF) [file pone.0187001.s001.pdf]
