# Supplementary material for: Clock-dependent and system-driven oscillators interact in the suprachiasmatic nuclei to pace mammalian circadian rhythms
Source: PLoS One. 2017 Oct 23;12(10):e0187001. doi: 10.1371/journal.pone.0187001 (PMC5653358; doi:10.1371/journal.pone.0187001)
Supplement: S1 Table — (DOCX) [file pone.0187001.s002.docx]

| **Gene** | **Forward** | **Reverse** |
| --- | --- | --- |
| *Per1* | CGGCCAGGTGTCGTGATTA | GGAGGACGAAACAGGGAAGG |
| *Per2* | GAGCAGTCTGCCAAAGCTGA | TCCTCATTAGCCTTCACCTGC |
| *Nr1d1* | GAGAGAGGCCATCACAACCT | GAGGAGCCACTAGAGCCAATG |
| *Dbp* | CTCGCCCACTTGGTACAGAA | GGGGGTTCAACCAGTCAGTT |
| *Hspa1a* | TTGTGTATTGCACGTGGGCT | GGGGCAGTGCTGAATTGAAG |
| *Odc1* | CTTCCATGTGGGCAGTGGAT | TTGACTGCAAGCGTGAAAGC |
| *Chordc1* | CCCAGATGAGCCAATGACAAA | GCTCCAATATTTCATCCCCTCGT |
| *Hprt2* | GCAGTACAGCCCCAAAATGG | GGTCCTTTTCACCAGCAAGCT |
| *Gapdh* | GGAGCGAGACCCCACTAACA | ACATACTCAGCACCGGCCTC |
| *Trfr1* | AGACCTTGCACTCTTTGGACATG | GGTGTGTATGGATCACCAGTTCCTA |
